# The efficacy of tofacitinib combined with bDMARDs in the treatment of ankylosing spondylitis patients with inadequate response to bDMARDs: a retrospective study

**DOI:** 10.1186/s41927-024-00373-y

**Published:** 2024-01-25

**Authors:** Jie Chang, Gang Wang

**Affiliations:** Department of Rheumatology, The Fourth Affiliated Hospital, Zhejiang University School of Medicine, N1 Shangcheng Road, 322000 Yiwu, Zhejiang province China

**Keywords:** Ankylosing spondylitis, Tofacitinib, JAK-inhibitor, Axial spondyloarthritis, bDMARD

## Abstract

**Introduction:**

Ankylosing spondylitis(AS) is a chronic inflammatory rheumatic disease primarily affecting the spine and sacroiliac joints. While biologic disease-modifying antirheumatic drugs(bDMARDs) and targeted synthetic DMARDs(tsDMARDs) are popular treatments for AS, there is limited research on their combined use. This study examined a cohort of AS patients who demonstrated inadequate response to bDMARDs and subsequently initiated combination therapy with tofacitinib in conjunction with bDMARDs, assessing both the efficacy and safety profile of this therapeutic approach.

**Methods:**

In this study, we retrospectively collected the electronic medical records (EMR) of 15 adult patients with AS who were admitted to the Fourth Affiliated Hospital Zhejiang University School of Medicine between January 2018 and June 2022. All patients had received at least one bDMARD treatment for more than three months and still exhibited moderate to high disease activity. Tofacitinib 5 mg bid was added to their original biological treatment. Treatment was continued for a minimum of 12 weeks following the initiation of combination therapy. Changes in ASDAS-CRP and BASDAI scores at week 12 were collected and analyzed from baseline, while changes in C-reactive protein (CRP) and erythrocyte sedimentation rate (ESR) at weeks 4, 8, and 12 were also collected and analyzed.

**Results:**

After 12 weeks of treatment, the overall ASDAS-CRP score decreased significantly from a baseline of 3.82 ± 1.47 (2.83 ~ 4.99) to 1.47 ± 0.48 (0.75 ~ 2.44), with remission achieved by 7 patients (46.7%) and low disease activity achieved by 5 patients (33.3%). The overall BASDAI score also showed significant improvement, decreasing from a baseline of 5.11 ± 1.42 (3.25 ~ 7 0.75) to 1.28 ± 0.70(0.20 ~ 2.55). Additionally, both ESR and CRP levels decreased significantly during the course of treatment without any reported adverse events leading to discontinuation.

**Conclusion:**

To a certain extent, our findings provide some evidence supporting the efficacy and safety of the combination of bDMARD and JAK inhibitor tofacitinib in AS patients with inadequate response to bDMARD monotherapy. It effectively controls disease activity while maintaining a relatively low and manageable incidence of adverse events. Further prospective randomized controlled trials with large sample sizes are anticipated to provide evidence-based medical support.

## Introduction

Ankylosing spondylitis (AS) is a chronic inflammatory rheumatic disease primarily affecting the spine and sacroiliac joints, resulting from an imbalance between innate and adaptive immune systems influenced by environmental factors [1]. AS induces inflammatory back pain that affects the spinal column and sacroiliac joints, leading to reduced quality of life for patients and increased societal burden [2].

Currently, the primary pharmacological interventions for AS encompass nonsteroidal anti-inflammatory drugs (NSAIDs), disease-modifying antirheumatic drugs (DMARDs), biologic agents (bDMARDs), and targeted synthetic DMARDs (tsDMARDs) [3]. BDMARDs primarily consist of tumor necrosis factor-α inhibitors (TNFi) such as etanercept, infliximab, adalimumab, and interleukin-17 A inhibitor (IL-17Ai) secukinumab [4]. TsDMARDs primarily consist of Janus kinase inhibitors (JAKi), such as tofacitinib, baritinib, and upadacitinib [5]. Biological agents and JAKi have demonstrated favorable efficacy in the treatment of AS and are increasingly favored therapeutic options. Nevertheless, a subset of patients remains unresponsive to bDMARDs or tsDMARDs. Additionally, the combination regimens utilized in AS primarily consist of NSAIDs or conventional synthetic DMARDs (csDMARDs) paired with a singular bDMARD or tsDMARD, whereas the employment of multiple bDMARDs or tsDMARDs possessing distinct mechanisms of action is infrequently documented [6].

This study retrospectively analyzed a cohort of AS patients with inadequate response to bDMARDs at the Fourth Affiliated Hospital Zhejiang University School of Medicine between January 2018 and June 2022, and subsequently initiated tofacitinib combination therapy on top of bDMARDs to assess its efficacy and safety in managing their disease.

## Methods

### Ethical statement

This retrospective study was approved by the ethics Committee of the Fourth Affiliated Hospital, Zhejiang University School of Medicine. All data were retrospectively collected and analyzed from complete patient records. Written informed consent was obtained from all enrolled patients.

### Study population

In this study, we retrospectively collected the electronic medical records (EMR) of adult patients (≥ 18 years old) with AS who were admitted to the Fourth Affiliated Hospital Zhejiang University School of Medicine from January 2018 to June 2022. The diagnosis of AS was based on either the modified New York criteria for AS in 1984 or the classification criteria for axial spondyloarthritis(axSPA) recommended by the Assessment of SpondyloArthritis International Society (ASAS) in 2009 [7]. All patients had received at least one bDMARD treatment for a duration of more than three months, and their disease activity remained moderate to high(Bath Ankylosing Spondylitis Disease Activity Index(BASDAI) ≥ 4 or Ankylosing Spondylitis Disease Activity Score(ASDAS-CRP) ≥ 2.1) [8]。.

Exclusion criteria: patients with severe illnesses, such as cancer or vital organ dysfunction (e.g. heart, lung, and kidney); co-infection; individuals who are unable to communicate effectively or have mental disorders; and incomplete EMR.

### Treatment modality

The preexisting biologics, including etanercept(25 mg twice a week), infliximab(6 mg/kg given at weeks 0, 2, and 6, followed by subsequent administrations every 8 weeks), adalimumab(40 mg once every two weeks), and secukinumab(150 mg once a week, and then every 4 weeks) were continued at the initiation of combination therapy in accordance with recommended doses and methods outlined in treatment guidelines and package inserts [9]. Concurrent use of NSAIDs or csDMARDs should be maintained. All patients received tofacitinib 5 mg twice daily in addition to their original biologics, and treatment was continued for at least 12 weeks after the initiation of combination therapy. The aforementioned medications were all included in the coverage of local medical insurance. In addition to pharmacological treatment, all patients were advised to implement non-pharmacological interventions such as smoking cessation, thermal insulation, and moderate exercise. However, due to the retrospective nature of the study and limitations in observation conditions, quantitative evaluation of these non-pharmacological interventions was not feasible.

### Outcome measurements

The primary outcome measures comprised the alteration in ASDAS-CRP score and BASDAI score at 12 weeks of treatment from baseline (1.3 ≤ ASDAS-CRP ≤ 2.1 defined as low disease activity, ASDAS-CRP ≤ 1.3 defined as remission). Secondary outcomes included the collection and analysis of C-reactive protein (CRP) and erythrocyte sedimentation rate (ESR) after 4, 8, and 12 weeks of treatment.

### Statistical analysis

The standard summary statistics were utilized to describe all parameters, including mean, standard deviation, minimum, and maximum. The paired Wilcoxon rank test was employed for intra-group comparisons before and after treatment while the independent rank sum test was used for between different groups. The statistical analysis was conducted using SPSS 27.0 software (IBM Corp., Armonk, NY), with a significance level of *P* < 0.05 set for all tests.

## Results

A total of 15 patients, consisting of 11 males and 4 females with an average age of 29.9 ± 6.72 (21–44) years, were enrolled in this study. Among the baseline bDMARDs administered, etanercept was used in four cases, infliximab in three cases, adalimumab in five cases, and secukinumab in three cases. Further baseline details are presented in Table 1.

### Primary outcome measurements

**Fig. 1:**
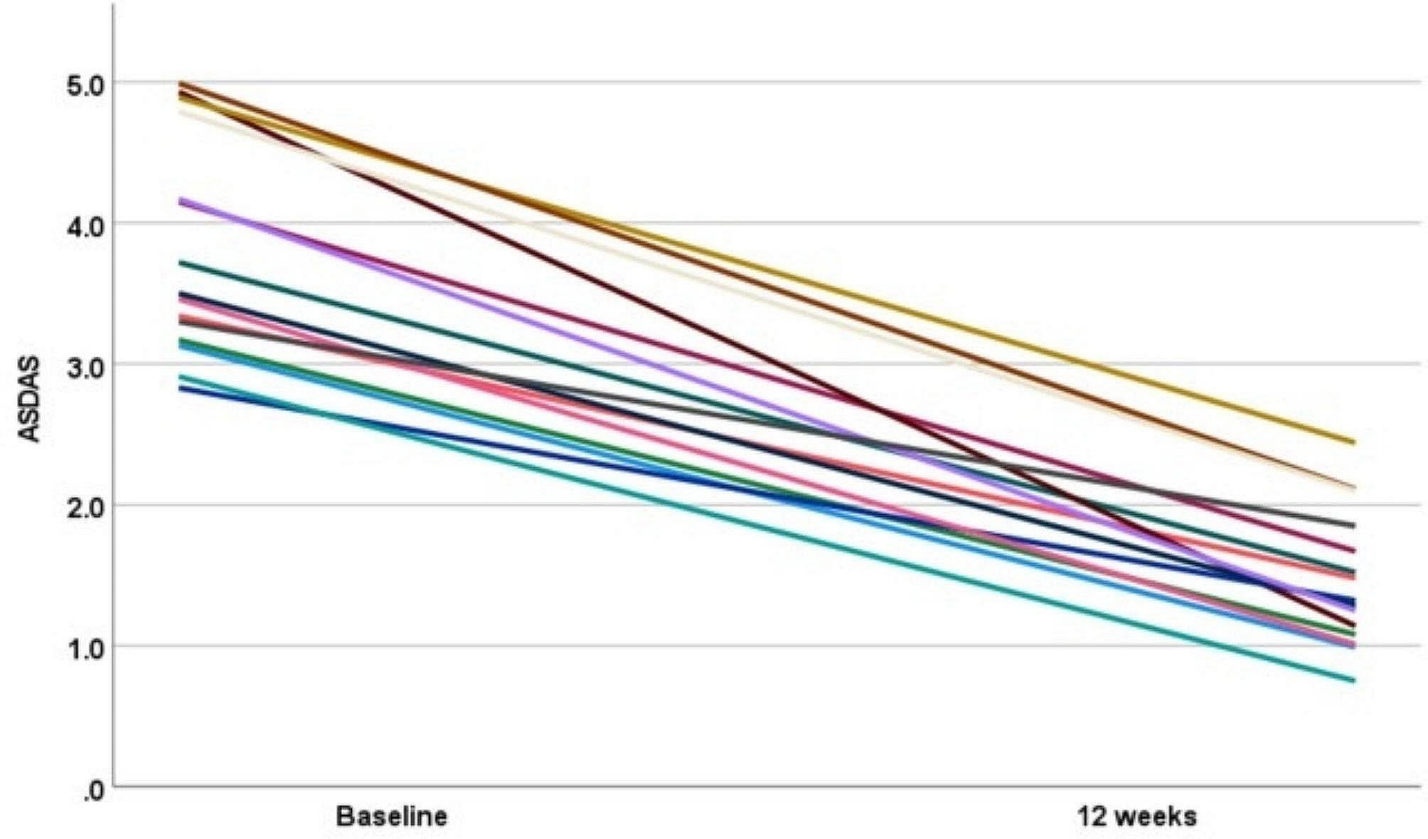
Line Chart of ASDAS changes at 0 and 12 weeks. ASDAS: Ankylosing Spondylitis Disease Activity Score

**Fig. 2:**
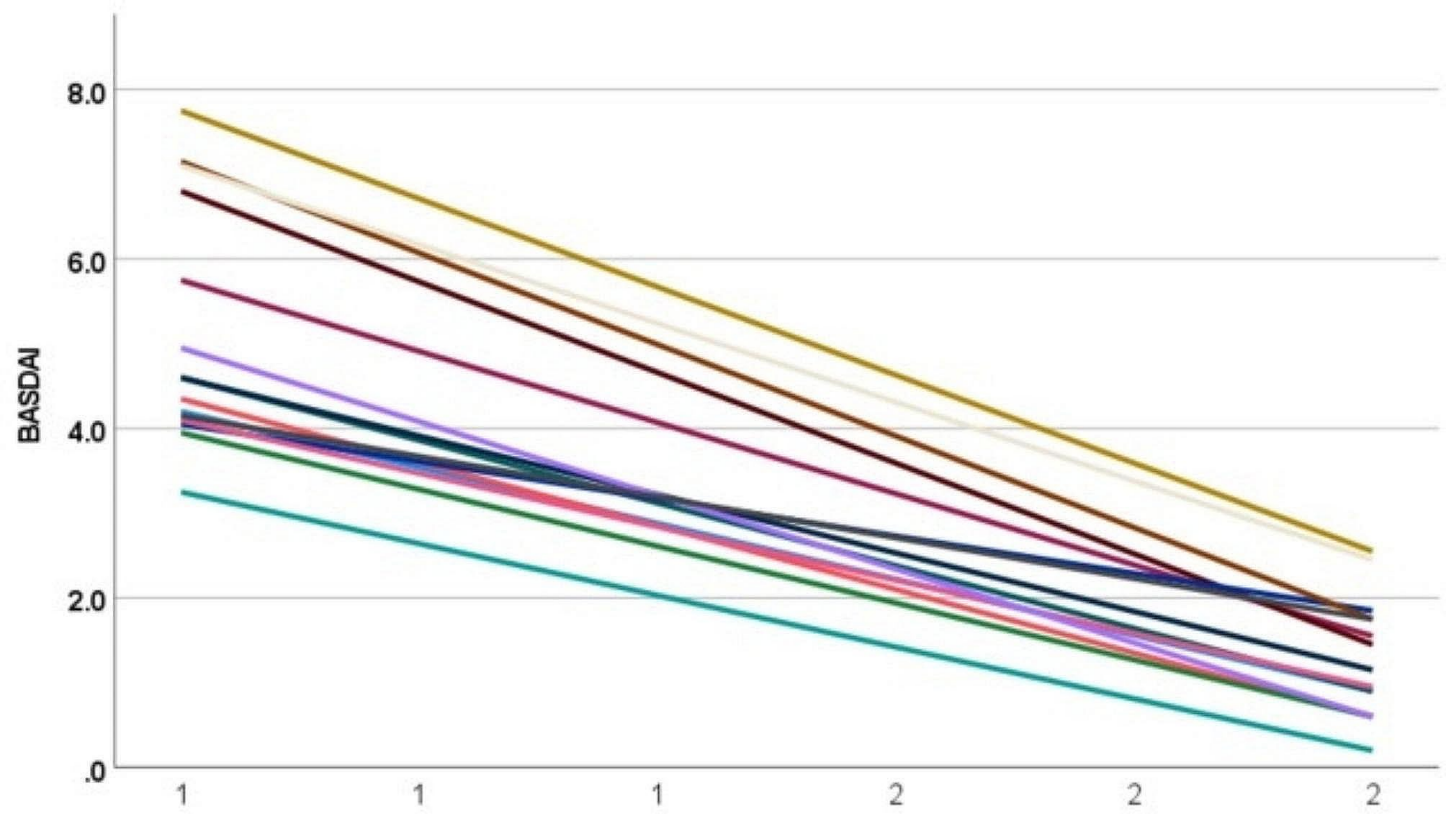
Line Chart of BASDAI changes at 0 and 12 weeks. BASDAI: Bath Ankylosing Spondylitis Disease Activity Index

After 12 weeks of treatment, the overall ASDAS-CRP score decreased significantly from a baseline of 3.82 ± 1.47 (2.83 ~ 4.99) to 1.47 ± 0.48 (0.75 ~ 2.44). Among them, remission (ASDAS-CRP < 1.3) was achieved in seven patients (46.7%), while low disease activity (1.3 ≤ ASDAS-CRP < 2.1) was observed in five patients (33.3%) (Table 2; Fig. 1). ASDAS-CRP decreased from 3.78 ± 0.72(3.13 ~ 4.79) to 1.41 ± 0.52(0.99 ~ 2.10) in the etanercept combined with tofacitinib group, from 4.22 ± 1.20(2.83 ~ 4.93) to 1.63 ± 0.70(1.14 ~ 2.44) in the infliximab group, from 3.99 ± 0.70(3.30 ~ 4.99) to 1.67 ± 0.33(1.25 ~ 2.11) in the adalimumab group, and from 3.19 ± 0.30(2.91 ~ 3.50) to 1.04 ± 0.27(0.75 ~ 1.28) in the secukinumab group, respectively. The overall BASDAI score exhibited a significant decrease from baseline (5.11 ± 1.42, range 3.25 to 7.75) to post-treatment (1.28 ± 0.70, range 0.20 to 2.55). Specifically, the etanercept combined with tofacitinib group showed a reduction from 5.00 ± 1.42 (4.10 ~ 7.10) at baseline to 1.30 ± 0.77(0.90 ~ 2.45), while the infliximab group demonstrated a decrease from 6.20 ± 1.92(4.05 ~ 7.75) to 1.95 ± 0.56(1.45 ~ 2.55). The adalimumab and secukinumab groups also experienced reductions in their BASDAI scores, with decreases observed from 5.27 ± 1.22(4.15 ~ 7.15) to 1.25 ± 0.60(0.60 ~ 1.75) and from 3.93 ± 0.68(3.25 ~ 4.60) to 0.65 ± 0.48(0.20 ~ 1.15), respectively (Table 2; Fig. 2).

### Secondary outcome measurements

**Fig. 3:**
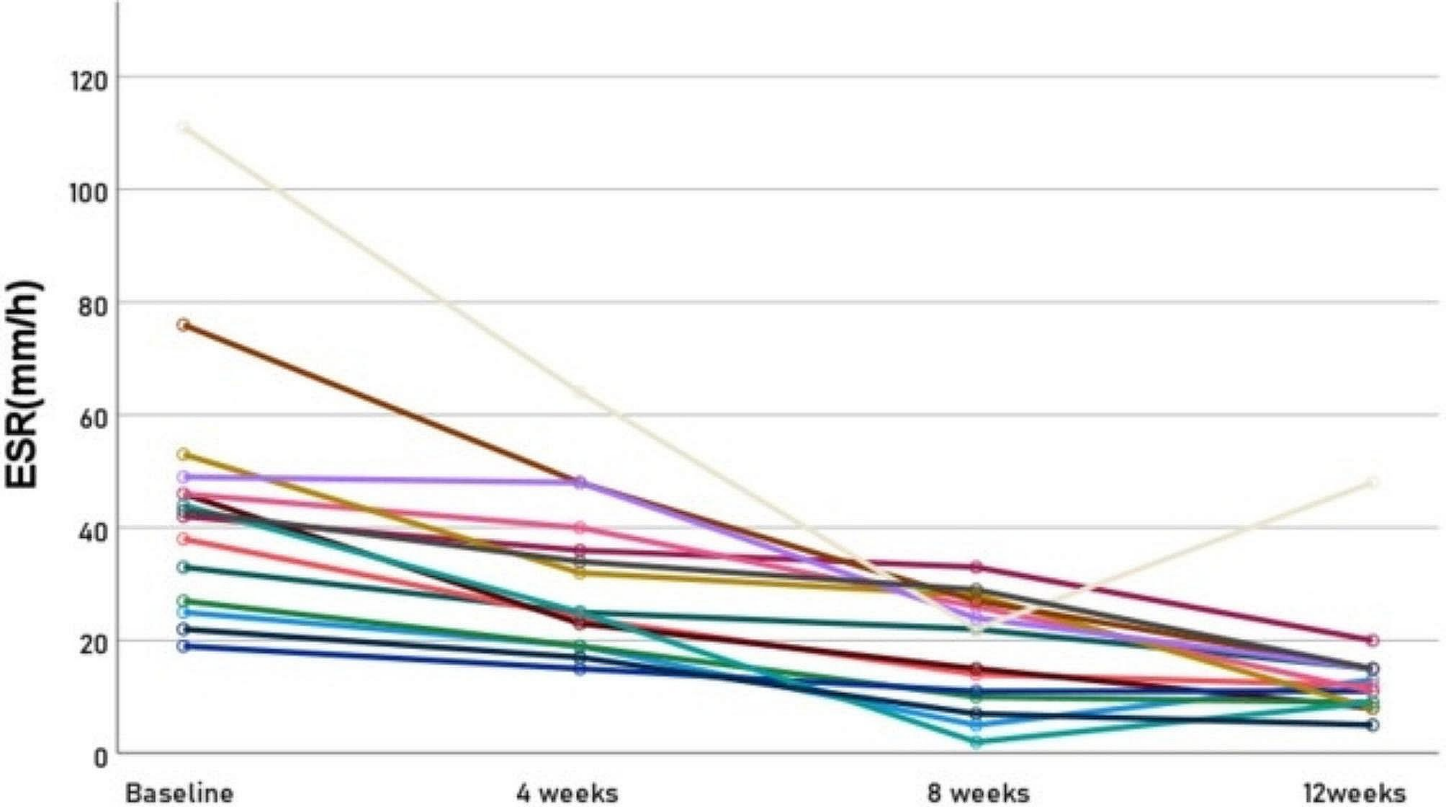
Line Chart of ESR changes at 0, 4, 8 and 12 weeks. ESR: erythrocyte sedimentation rate

**Fig. 4:**
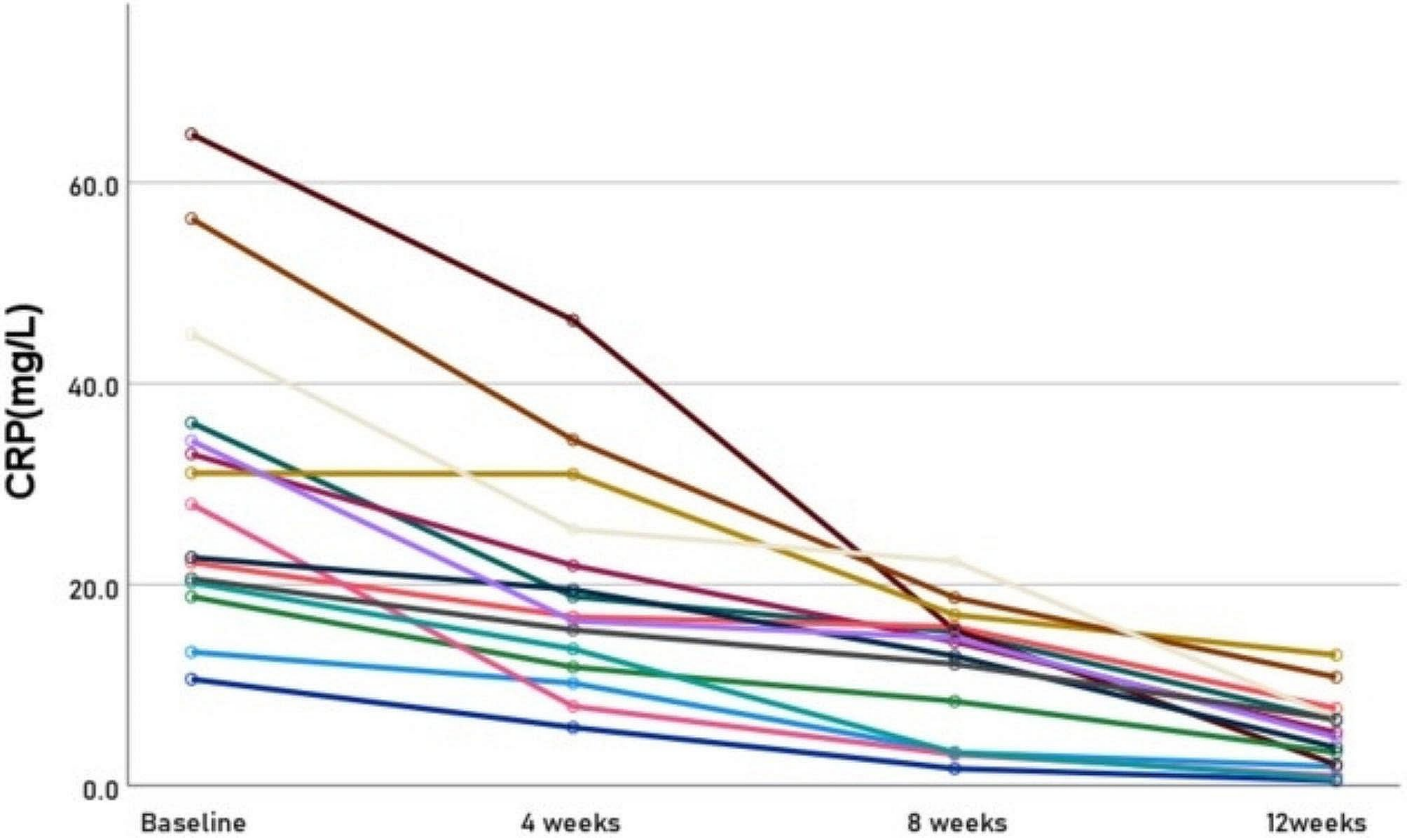
Line Chart of CRP changes at 0, 4, 8 and 12 weeks. CRP: C-reactive protein

At 4, 8, and 12 weeks of treatment, ESR decreased from 44.93 ± 23.20(19 ~ 111) at baseline to 31.27 ± 13.91(15 ~ 64) at 4 weeks, 18.33 ± 9.79(2 ~ 33) at 8 weeks, and 14.27 ± 10.07(5 ~ 48) at 12 weeks, with significant differences from baseline at 8 and 12 weeks (Fig. 3). CRP decreased from 30.47 ± 15.30(10.6 ~ 64.8) at baseline to 30.47 ± 15.30(10.6 ~ 64.8) at 4 weeks, to 11.89 ± 6.42(1.7 ~ 22.3) at 8 weeks, to 4.99 ± 3.69(0.5 ~ 13.0) at 12 weeks, all significantly different from baseline (Fig. 4). The specific data are detailed in Table 2.

### Adverse reactions

Two patients, one receiving baseline etanercept and the other secukinumab, experienced mild stomach discomfort upon addition of tofacitinib. A female patient treated with adalimumab in combination with tofacitinib reported a mild upper respiratory tract infection that was resolved through symptomatic treatment. No patients discontinued treatment due to adverse events.

## Discussion

AS is a prevalent rheumatic disease that primarily affects young individuals. If left uncontrolled, it can lead to spinal and sacroiliac joint destruction and fusion, significantly impacting patients’ quality of life. Consequently, patient treatment needs and expectations are generally high. As per the updated 2022 ASAS-EULAR recommendations for managing axial spondyloarthritis, patients with persistent high disease activity despite conventional therapy should be considered for TNF inhibitors, IL-17 inhibitors or JAK inhibitors. Current practice suggests starting with TNF-α or IL-17 inhibitors. If treatment fails with the first bDMARDs or tsDMARDs, switching to another bDMARDs or JAK inhibitor can be considered [10].

For patients with AS who exhibit inadequate response to NSAIDs or bDMARDs, there have been numerous reports indicating the efficacy of switching to JAKi [11]. Overall response rates, such as ASAS20 response rate, range from 42–60% [12–14]. At our center, many patients have switched to JAKi due to insufficient responses to bDMARDs; some experienced improved disease outcomes while others exhibited inadequate responses solely on JAKi therapy. Therefore, this study attempted to combine bDMARDs with JAKi. The results indicate that the majority of patients exhibited a favorable response, as evidenced by significant reductions in ESR and CRP after four weeks of treatment (as depicted in Figs. 3 and 4). These findings suggest that the combination therapy may confer superior disease control compared to monotherapy with either agent alone.

There is limited literature on the concomitant use of bDMARDs and tsDMARDs, with a primary concern being the potential for adverse events such as serious infections, cardiovascular complications, thrombotic events, and malignancies [15, 16]. Therefore, we cautiously administered tofacitinib to a subset of patients who exhibited inadequate response to bDMARDs, resulting in the limited sample size of our study. In this study, the combination of bDMARDs and the JAK inhibitor tofacitinib demonstrated a lower incidence of adverse events, with only two cases of mild gastrointestinal discomfort and one case of mild upper respiratory tract infection observed. This may be related to the shorter observation time, younger age, and better basic constitution of the patients. These findings suggest that the combination therapy may be relatively safe in certain patient populations, although caution should be exercised when considering long-term use.

Our study is subject to several limitations. Firstly, the results and findings may be influenced by confounding factors such as patients’ lifestyle habits, comorbidities, and medications administered for reasons other than bDMARDs. The nonpharmacologic interventions, such as smoking cessation, maintaining warmth, and engaging in adequate exercise, were recommended for each patient [17]. However, due to the objective local conditions, subjective compliance of patients, and the retrospective nature of the study design, quantifying the effectiveness of these interventions was not feasible. None of these potential confounding factors could be definitively excluded. Secondly, due to the small sample size and limited data on short-term treatment duration available, we were unable to obtain long-term outcomes or observe additional adverse events that may arise from prolonged use. A control group was not included additionally. Thirdly, this retrospective study lacked strict inclusion and exclusion criteria, potentially leading to selection bias, and lacked a control group, resulting in low levels of evidence-based support. Additionally, all data was sourced from Fourth Affiliated Hospital Zhejiang University School of Medicine as a single center, which may limit generalizability to other hospitals.

In conclusion, to a certain extent, our findings provide some evidence supporting the efficacy and safety of the combination of bDMARD and JAK inhibitor tofacitinib in AS patients with inadequate response to bDMARD monotherapy. It effectively controls disease activity while maintaining a relatively low and manageable incidence of adverse events. Further prospective randomized controlled trials with large sample sizes and a meticulously designed protocol are anticipated to provide evidence-based medical support.

**Table 1:** Baseline information of patients. ETN: etanercept; ADA: adalimumab; INF: infliximab; SEC: secukinumab

Patient number	Sex	Age	Baseline bDMARD	Baseline ESR(mm/h)	Baseline CRP(mg/L)	Baseline BASDAI	Baseline ASDAS-CRP
1	Female	31	ETN	25	13.3	4.20	3.13
2	Male	25	ETN	33	36.1	4.60	3.72
3	Female	28	ADA	42	33.0	5.75	4.15
4	Female	22	ADA	38	22.2	4.35	3.34
5	Male	21	INF	46	64.8	6.80	4.93
6	Male	37	SEC	27	18.8	3.95	3.17
7	Male	30	INF	19	10.6	4.05	2.83
8	Male	42	ETN	46	28.0	4.10	3.46
9	Male	28	INF	53	31.1	7.75	4.89
10	Female	33	SEC	44	20.2	3.25	2.91
11	Male	26	SEC	22	22.7	4.60	3.50
12	Male	44	ADA	76	56.4	7.15	4.99
13	Male	33	ADA	49	34.3	4.95	4.17
14	Male	23	ETN	111	44.9	7.10	4.79
15	Male	26	ADA	43	20.6	4.15	3.30

**Table 2:** Changes of BASDAI, ASDAS-CRP, ESR and CRP

		BASDAI	ASDAS-CRP	ESR(mm/h)	CRP(mg/L)
**All patients**	**Baseline**	5.11 ± 1.42(3.25 ~ 7.75)	3.82 ± 1.47(2.83 ~ 4.99)	44.93 ± 23.20(19 ~ 111)	30.47 ± 15.30(10.6 ~ 64.8)
	**Week 4**			31.27 ± 13.91(15 ~ 64)	19.69 ± 10.86(5.8 ~ 46.3)
	**Week 8**			18.33 ± 9.79(2 ~ 33)	11.89 ± 6.42(1.7 ~ 22.3)
	**Week 12**	1.28 ± 0.70(0.20 ~ 2.55)	1.47 ± 0.48(0.75 ~ 2.44)	14.27 ± 10.07(5 ~ 48)	4.99 ± 3.69(0.5 ~ 13.0)
	**P(week 4, 8, 12 vs. Baseline)**	**< 0.001**	**< 0.001**	0.060, **< 0.001, < 0.001**	**0.034, < 0.001, < 0.001**
**Etanercept group**	**Baseline**	5.00 ± 1.42(4.10 ~ 7.10)	3.78 ± 0.72(3.13 ~ 4.79)	53.75 ± 39.14(25 ~ 111)	30.58 ± 13.43(13.3 ~ 44.9)
	**Week 4**			37.00 ± 20.05(19 ~ 64)	15.60 ± 8.10(7.9 ~ 25.5)
	**Week 8**			18.75 ± 9.36(5 ~ 26)	11.00 ± 9.45(3.1 ~ 22.3)
	**Week 12**	1.30 ± 0.77(0.90 ~ 2.45)	1.41 ± 0.52(0.99 ~ 2.10)	21.75 ± 17.58(11 ~ 48)	4.05 ± 3.03(1.0 ~ 6.8)
	**P(week 4, 8, 12 vs. Baseline)**	**0.007**	**0.002**	0.475, 0.133, 0.186	0.105, 0.054, **0.008**
**Infliximab group**	**Baseline**	6.20 ± 1.92(4.05 ~ 7.75)	4.22 ± 1.20(2.83 ~ 4.93)	39.33 ± 17.95(19 ~ 53)	35.50 ± 27.37(10.6 ~ 64.8)
	**Week 4**			23.33 ± 8.50(15 ~ 32)	27.70 ± 20.45(5.8 ~ 46.3)
	**Week 8**			18.00 ± 8.89(11 ~ 28)	11.40 ± 8.43(1.7 ~ 17.0)
	**Week 12**	1.95 ± 0.56(1.45 ~ 2.55)	1.63 ± 0.70(1.14 ~ 2.44)	9.00 ± 1.73(8 ~ 11)	5.20 ± 6.80(0.5 ~ 13.0)
	**P(week 4, 8, 12 vs. Baseline)**	**0.021**	**0.032**	0.235,0.139, 0.098	0.713, 0.219, 0.136
**Adalimumab group**	**Baseline**	5.27 ± 1.22(4.15 ~ 7.15)	3.99 ± 0.70(3.30 ~ 4.99)	49.60 ± 15.27(38 ~ 76)	33.30 ± 14.31(20.6 ~ 56.4)
	**Week 4**			38.00 ± 10.20(24 ~ 48)	21.00 ± 7.90(15.5 ~ 34.4)
	**Week 8**			25.40 ± 7.16(14 ~ 33)	15.12 ± 2.41(12.1 ~ 18.7)
	**Week 12**	1.25 ± 0.60(0.60 ~ 1.75)	1.67 ± 0.33(1.25 ~ 2.11)	15.40 ± 2.88(12 ~ 20)	7.04 ± 2.39(4.8 ~ 10.8)
	**P(week 4, 8, 12 vs. Baseline)**	**< 0.001**	**< 0.001**	0.196, **0.012, 0.001**	0.131, **0.023**, **0.004**
**Secukinumab group**	**Baseline**	3.93 ± 0.68(3.25 ~ 4.60)	3.19 ± 0.30(2.91 ~ 3.50)	31.00 ± 11.53(22 ~ 44)	20.57 ± 1.98(18.8 ~ 22.7)
	**Week 4**			20.33 ± 4.16(17 ~ 25)	14.97 ± 4.03(11.8 ~ 19.5)
	**Week 8**			6.33 ± 4.04(2 ~ 10)	8.20 ± 4.80(3.3 ~ 12.9)
	**Week 12**	0.65 ± 0.48(0.20 ~ 1.15)	1.04 ± 0.27(0.75 ~ 1.28)	7.67 ± 2.31(5 ~ 9)	2.60 ± 1.66(0.7 ~ 3.8)
	**P(week 4, 8, 12 vs. Baseline)**	**0.002**	**< 0.001**	**0.206, 0.025, 0.026**	0.097, **0.015**, **< 0.001**

## Data Availability

The data that support the findings of this study are available on request from the corresponding author Gang Wang, upon reasonable request.
